# The carbon footprint of tourism businesses in Pavlodar region (Kazakhstan): Baseline assessment and decarbonization hot spots

**DOI:** 10.1371/journal.pone.0338937

**Published:** 2025-12-11

**Authors:** Dinara Yessimova, Alexandr Belyy, Alina Faurat, Aleksandra Novikova-Rodi, Marina Olshanskaya, Liliya Zavyalova, Iuliia Grishchenko, Ayana Yessim

**Affiliations:** 1 Department of Geography and Tourism, Toraighyrov University, Pavlodar, Republic of Kazakhstan; 2 Institute for Climate Protection, Energy and Mobility (IKEM), Berlin, Germany; 3 AvantGarde Group, Bratislava, Slovakia; 4 AvantGarde Central Asia, Tashkent, Uzbekistan; 5 The International Institute for Industrial Environmental Economics, Lund University, Lund, Sweden; 6 L.N. Gumilyov Eurasian National University, Department of Tourism, Astana, Republic of Kazakhstan; Wuhan University of Technology, CHINA

## Abstract

The study presents the first detailed carbon footprint assessment of tourism businesses in Kazakhstan’s Pavlodar region, aiming to establish a baseline and identify decarbonization pathways that align with the country’s 2060 carbon neutrality target. Using the GHG Protocol methodology, we calculated the footprint for two tourism properties in the Bayanaul district: a year-round holiday home and a seasonal summer hotel. Two pilot sites were selected to be representative of the region’s tourism businesses, exhibiting distinct operational and energy consumption patterns. The analysis revealed distinct emission profiles and significant contributions from indirect sources. The year-round facility, reliant on coal for heating, generated a total of 529 tCO_2_-eq. in З, whereas the seasonal hotel produced 185 tCO_2_-eq. A critical finding for both businesses was the dominance of Scope 3 emissions, which accounted for 57% to 62% of their total footprints, primarily driven by tourist transportation, waste generation, and food procurement. Scope 1 emissions from on-site fuel combustion were significant only for the year-round property, while Scope 2 emissions from purchased electricity were a key factor for both. Based on these findings and guided by the Science-Based Targets initiative (SBTi) and Kazakhstan’s national strategies, such as the Concept for Transition to Green Economy and the Strategy for Achieving Carbon Neutrality by 2060, we propose a decarbonization roadmap. The targets include an absolute emissions reduction of 15% by 2030, 25% by 2040, and 50% by 2050. Key recommendations include phasing out coal by 2050, transitioning to low-carbon electricity by 2035, and implementing strategies to mitigate value chain emissions, including those associated with transportation, nutrition, and waste. This research provides a replicable framework for tourism operators in Kazakhstan and similar regions to measure and manage their environmental impact, contributing to a more sustainable tourism sector.

## 1. Introduction

**Global context.** The latest Intergovernmental Panel on Climate Change (IPCC) Synthesis Report underscores the urgency of limiting warming to 1.5 °C, which implies deep, near-term cuts and achieving global net-zero greenhouse gas (GHG) emissions around mid-century [1]. Tourism is part of this collective effort: it both contributes to GHG emissions and is increasingly exposed to climate risks. International initiatives—such as the Glasgow Declaration on Climate Action in Tourism—call for halving emissions by 2030 and reaching net-zero by 2050, providing a sector-wide framing for mitigation pathways and accountability [2]. At a global scale, tourism’s carbon footprint has been estimated at 8% of total GHG emissions when supply-chain effects are included [3], emphasizing the importance of decarbonizing both direct operations and value chains.

**Problem statement (Kazakhstan/Pavlodar energy context).** In Kazakhstan, and particularly in Pavlodar region, decarbonization challenges are shaped by a coal-dependent energy mix and a continental climate with pronounced heating and cooling needs. These structural factors make the energy component of tourism businesses—Scopes 1–2 under the GHG Protocol—especially critical: on-site fuel use for space/water heating and electricity consumed from a carbon-intensive grid can dominate operational footprints, while also influencing upstream emissions and downstream service profiles. Understanding these energy-related drivers is a prerequisite for setting realistic, place-appropriate reduction targets and for sequencing actions that align with national long-term climate objectives.

**Knowledge gap.** Although several studies have examined aspects of sustainable tourism development in Pavlodar region—including sector readiness and environmental performance [4–6]—there is, to our knowledge, no published, quantitative, business-level carbon footprint assessment for local tourism enterprises that decomposes emissions by scopes and value-chain categories. This constitutes a clear gap for evidence-based policymaking, benchmarking, and project-level planning.

**Aim and objectives.** The aim of this study is to establish a transparent baseline of GHG emissions for typical tourism businesses in Pavlodar region and to identify decarbonization hot spots aligned with international best practice and national priorities. To achieve this aim, we:

- quantify Scopes 1, 2, and 3 emissions for two representative enterprises using GHG Protocol–compatible methods;
- analyze the structure of emissions (by scope and category) to locate operational and value-chain hot spots;
- benchmark results against ranges reported in international literature and initiatives relevant to tourism decarbonization [2,3,7–11];
- formulate practical recommendations and near- to long-term targets for energy supply and demand (including coal phase-down and low-carbon electricity);
- outline opportunities for complementary measures (e.g., waste, procurement, mobility) and discuss options for offsets and measurement-reporting-verification (MRV) to support continuous improvement.

**Contribution and article structure.** The study offers (i) the first quantitative, scope-resolved carbon footprint at the level of tourism businesses in Pavlodar region, filling a documented evidence gap for Kazakhstan; and (ii) a decision-oriented framework that links diagnostic results to actionable targets consistent with international tourism climate commitments [2,3] and the broader literature on accommodation-sector footprints [7–10]. The remainder of the paper is organized as follows: Section 2 details the methodology; Section 3 presents the results; Section 4 provides discussion and interpretation; Section 5 concludes.

**Fig 1:**
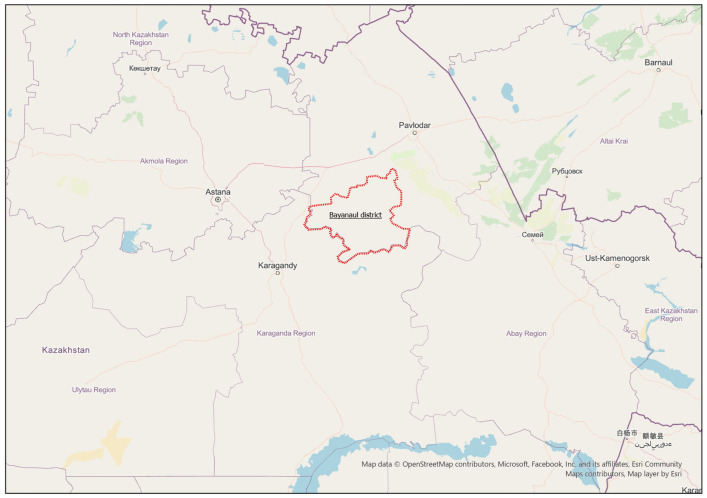
Pavlodar region and Bayanaul region (highlighted in red) on the map of Kazakhstan *(Contains the information from OpenStreetMap and OpenStreetMap Foundation, which is made available under the Open Database License).*

The Pavlodar region is area in Kazakhstan with considerable potential for tourism, owing to its rich historical, cultural and natural assets. In 2023, the region attracted over 100 thousand domestic and foreign tourists, the majority of whom visited Bayanaul National Park (Fig 1). This figure represents approximately 1.5% of the national tourist influx and is composed primarily of local and cross-boarder visitors from Russia. While over half of the region’s tourism businesses operate year-round, peak summer seasons see occupancy rates reach 100%, resulting in significant environmental pressure on local ecosystems.

Therefore, this paper is the first known attempt to calculate GHG emissions from two typical tourism business in Pavlodar region of Kazakhstan. This case study serves as a foundational step to estimate GHG emissions of the region’s entire tourism sector. While global research has documented the significant carbon footprint of the tourism sector focusing on such aspects as transportation, accommodation, and activities, this study provides a detailed and localized analysis for a region in Kazakhstan, characterized with unique challenges related to its energy mix and local specifics. The findings contribute vital, context-specific data to the broader understanding of tourism’s environmental impact, particularly within a developing economy transitioning towards sustainability.

## 2. Methodology overview

### 2.1. Framework and scopes (summary)

The carbon footprint (CF) is the total greenhouse gas (GHG) emissions attributable to an activity or organization over a defined period, typically expressed in CO₂-equivalent. In this study, CF is used as a decision-oriented indicator to diagnose emission “hot spots” at tourism businesses and to enable consistent tracking against local decarbonization goals.

We compiled the inventory by integrating widely used standards for GHG accounting in tourism and related sectors—principally the GHG Protocol and sector guidance—together with IPCC methodologies and practice-oriented manuals for tourism operations [12–17]. This combination ensures transparent scope setting, comparable calculations, and compatibility with international reporting.

**Fig 2:**
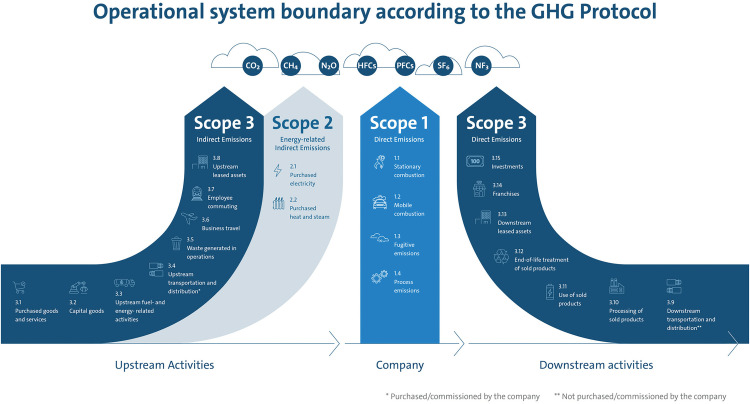
Three Scopes of GHG emissions under the GHG Protocol (https://ghgprotocol.org/corporate-standard) [18].

Under the GHG Protocol, emissions are grouped into three scopes that reflect ownership or control and the locus of energy use (see Fig 2):

- Scope 1 (direct): on-site fuel combustion and other direct process emissions from sources owned or controlled by the facility.
- Scope 2 (energy-indirect): emissions from the generation of purchased electricity, steam, heat, or cooling consumed by the facility [18].
- Scope 3 (other indirect): upstream and downstream emissions not captured in Scopes 1–2 (e.g., purchased goods and services, waste, business travel, and visitor transport associated with the service).

### 2.2. System boundaries and data sources

The simplest method to calculate the tourism sector footprint is to conduct these calculations at the level of an individual business. This approach was applied in several academic publications [7–10], so as was it applied also in our study. The organizational boundary comprises two representative tourism businesses in Pavlodar region (Holiday Home “A” and Hotel “B”) (The names of the pilot properties were changed at the request of the owners in order to maintain the confidentiality of the information received).

The operational boundary includes direct fuel use (Scope 1), purchased electricity/heat (Scope 2), and material upstream/downstream categories material to the case context (Scope 3). The reporting period is calendar year 2022, serving as the baseline for subsequent tracking. Primary data were obtained from facility records (utility bills, fuel logs, procurement data) and short structured interviews; secondary data include national statistics and published databases.

### 2.3. Calculation approach

***Emission factors***. Emission factors (EFs) convert activity data to CO₂-equivalents using recognized national and international sources and benchmarks; for Kazakhstan we rely on available factors and regulatory benchmarks for regulated sectors [19]. Where a Pavlodar-specific factor was not available (notably for local coal), we derived a local EF using the standard calorific-value-based method:


$${EF}_{coal}=\frac{LCV\;\times \;{EF}_{fuel}\times GWP}{OF\times {\text{1}\text{0}}^{\text{6}}}$$ (1)

where, according to the supporting materials of the ‘Methodologies for calculating GHG emissions and absorption’ adopted by the Ministry of Ecology and Natural Resources of the Republic of Kazakhstan:

- ${EF}_{coal}$ is the emission factor for coal (kgCO_2_-eq./t)
- LVC is the lower calorific value (0.0119 TJ/t)
- ${EF}_{fuel}$ is the fuel-specific emission factor (101 kg/TJ or 0.101 t/TJ)
- GWP is the global warming potential (1.0 for CO₂)
- OF is the carbon oxidation factor (1.0 for CO_2_).

Based on this calculation, the emission factor for local coal, which was determined to be 1201.9 kgCO₂-eq/t.

***Calculation method.*** GHG emissions are calculated as *activity data × EF*, aggregated and reported as CO₂-equivalents; categories are organized by Scopes 1–3 in line with the GHG Protocol (see Fig 2). The basic relationship used throughout is:


$$\text{GHG}\;\text{emissions}\;\left({tCO}_{\text{2}-}\text{eq}.\right)=\;\text{Activity}\;\text{data}\;(\text{activity}\;\text{unit})\text{x}\;\text{Emission}\;\text{factor}\;\left(\frac{\text{tCO2}-\text{eq}.}{\text{activity}}\text{unit}\right)$$ (2)

All entries were reconciled to the organizational/operational boundaries and reporting period. Scopes 1–2. Metered fuel use and purchased electricity/heat were taken directly from facility records and converted to CO₂-eq using category-appropriate emission factors (regional grid factor for Pavlodar; locally derived coal EF from (1)). Results are organized by the GHG Protocol scopes (see Fig. 2) and aggregated with (2).

Scope 3 (material categories only). We quantified only categories material to the facilities’ profiles, using (2) and the best available activity data:

- Category 1: Purchased goods & services (food) — mass of item groups with database EFs; illustrative per-guest scaling uses.
- Category 3: Fuel/electricity life cycle — upstream (well-to-tank) additions consistent with the local energy mix.
- Category 4: Upstream transport — distance/activity by mode (passenger-km/ton-km) with modal EFs.
- Category 5: Waste — annual mass by type with disposal/treatment EFs (landfill/wastewater). The footprint was calculated by multiplying the total annual mass of solid waste and volume of wastewater produced by each hotel by the relevant emission factors for landfill disposal and water treatment, sources from DEFRA.

## 3. Calculation results

### 3.1. 2022 GHG emission inventory

The results of the GHG inventory for the 2022 baseline year for both properties are presented in Tables 1 and 2. The total carbon footprints were calculated to be 529 tCO_2_-eq. for Holiday Home “A” and 185 tCO_2_-eq. for Hotel “B”.

**Table 1:** Greenhouse gas inventory results (tons of CO_2-_eq.) for Scopes 1, 2, and 3, for Holiday Home «A».

GHG sources	2022	% of Scope
**Total (Scope 1 + Scope 2)**	**227**	
**Total (Scope 1 + Scope 2 + Scope 3)**	**529**	
**Scope 1**	**108**	**20**
Mobile sources	–	
Stationary sources	108	
**Scope 2**	**119**	**23**
Commercial/purchased electricity, market method	119	
**Scope 3**	**302**	**57**
Category 1: Purchased goods and services	53	
Category 3: Fuel and electricity life cycle (emissions not included in Scopes 1 or 2)	52	
Category 4: Upstream transportation	107	
Category 5: Waste generated	90	

**Table 2:** Greenhouse gas inventory results (tons of CO_2-_eq.) for Scopes 1, 2, and 3, for Hotel «B».

GHG sources	2022	% of Scope
**Total (Scope 1 + Scope 2)**	**70**	
**Total (Scope 1 + Scope 2 + Scope 3)**	**185**	
**Scope 1**	**2**	**1**
Mobile sources	2	
Stationary sources	–	
**Scope 2**	**68**	**37**
Commercial/purchased electricity, market method	68	
**Scope 3**	**115**	**62**
Category 1: Purchased goods and services	26	
Category 3: Fuel and electricity life cycle (emissions not included in Scopes 1 or 2)	7	
Category 4: Upstream transportation	58	
Category 5: Waste generated	24	

A key finding for both businesses is the dominance of Scope 3 emissions, which account for 57% of the total footprint for Holiday Home “A” and 62% for Hotel “B”. Within Scope 3, emissions from tourist transportation (Category 4) and waste (Category 5) were the most significant contributors.

The emission profiles for Scope 1 and Scope 2 differ notably between the two properties, primarily shaped by their energy sources and operational models. Holiday Home “A” is heavily reliant on the direct combustion of hard coal for heating, making stationary combustion its primary source of Scope 1 emissions (108 tCO_2_-eq.). In contrast, Hotel “B”, operating mainly in summer, has negligible heating requirements and its Scope 1 emissions are minimal. Instead, its footprint is dominated by Scope 2 emissions from its consumption of grid electricity (68 tCO_2_-eq.), which, given the carbon intensity of Kazakhstan’s national grid, represents a significant source of indirect emissions.

### 3.2. Proposed decarbonization targets

Based on the 2022 inventory, science-based decarbonization targets were established for each tourism business. Targets are considered “science-based” (SBT) if they align with emissions reduction trajectory required to limit global warming to 1.5°C above pre-industrial levels, as outlined in the Paris Agreement. Therefore, we applied the framework and the level of ambition of the Science Based Targets initiative (SBTi), including those outlines in SBTi’s recommendations and standards, including SBTi, 2023a, 2023b, 2023c [20].

The type of sectoral activity determines the applicable target and ambition level. According to the indicated SBTi recommendations, general economic activities should follow the 1.5°C pathway in Scope 1 and Scope 2, and at least the 2.0°C pathway in Scope 3, in line with the IPCC and IEA scenarios. The targets should follow a linear reduction approach that results in a 42% emission reduction by 2030 in Scope 1 and 2 and a 27% emission reduction in Scope 3.

The proposed decarbonization targets were further contextualized by considering Kazakhstan’s national climate policies. These included the Concept for Transition of the Republic of Kazakhstan to Green Economy, the Nationally Determined Contribution (NDC) of Kazakhstan, the Strategy for Achieving Carbon Neutrality of the Republic of Kazakhstan until 2060, as well as the Concept for the Development of Energy Saving and Energy Efficiency in the Republic of Kazakhstan for 2023–2029.

Short/medium- and long-term targets were developed for each scope. Furthermore, we suggested setting decarbonization targets for specific energy carriers, namely coal and electricity, in addition to scope targets. Tables 3 and 4 present the developed decarbonization targets for tourism businesses A and B.

**Table 3:** Proposed targets for Scope 1 and 2 emission reduction in pilot tourism businesses.

	Holiday Home «A»	Hotel «B»
Base year	2022	2022
Base year emissions	•Scope 1: 108 tons of CO_2_-eq. •Scope 2: 119 tons of CO_2_-eq.	•Scope 1: 2 tons of CO_2_-eq. •Scope 2: 68 tons of CO_2_-eq.
2030 target	•Decrease absolute emissions by 15%	•Decrease absolute emissions by 15%
2040 target	•Decrease absolute emissions by 25%	•Decrease absolute emissions by 25%
2050 target	•Decrease absolute emissions by 50%	•Decrease absolute emissions by 50%
Additional targets	•Transition to low-carbon electricity by 2035 •Complete substitution of coal by other energy sources by 2050.	•Transition to low-carbon electricity by 2035
Rationale	•Alignment to the SBTi recommendations and standards •Analysis by the German Institute for Economic Research (DIW-Econ) and GIZ on Kazakhstan’s carbon neutrality •The International Energy Agency’s Net Zero by 2050 by Scenario •The Glasgow Declaration on Climate Action in Tourism •Consideration of Kazakhstan’s national climate strategies	

**Table 4:** Proposed targets for Scope 3 emission reduction in pilot tourism businesses.

	Holiday Home «A»	Hotel «B»
Base year	2022	2022
Base year emissions	•Scope 3: 302 tons of CO2eq.	•Scope 3: 115 tons of CO2eq.
2027 target: Waste (Category 5)	•Develop a concept for sustainable waste management.	
2027 target: Procurement (Category 1)	•Develop a concept for sustainable nutrition and procurement, including a higher share of plant-based foods in the diet and a higher share of local products in procurement	
2028 target	•Implementation of the developed concepts.	
Rationale	•Alignment to the SBTi recommendations and standards •Consideration of Kazakhstan’s national climate strategies	

## 4. Discussion and interpretation

**Fig 3:**
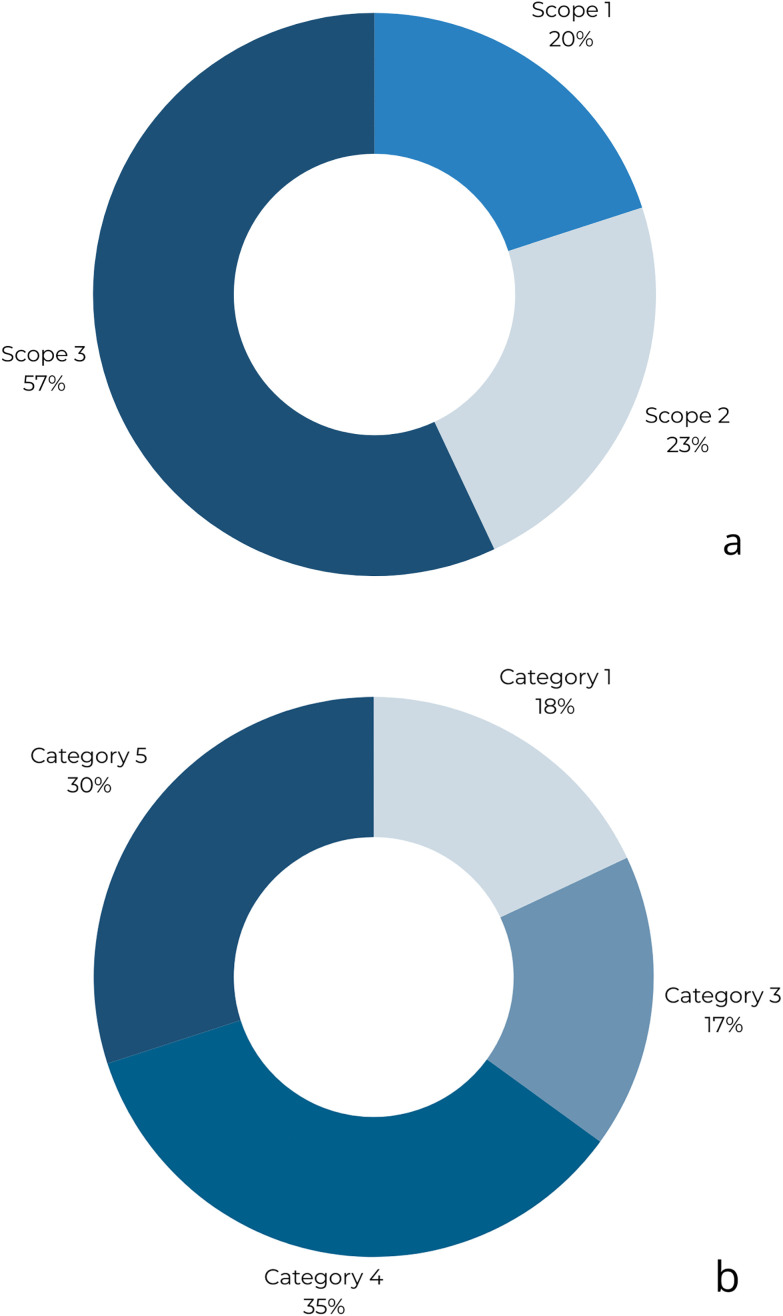
GHG emissions (as %) by Scopes (a) and composition of GHG emissions (as %) under Scope 3 (b) for Holiday Home «A».

**Fig 4:**
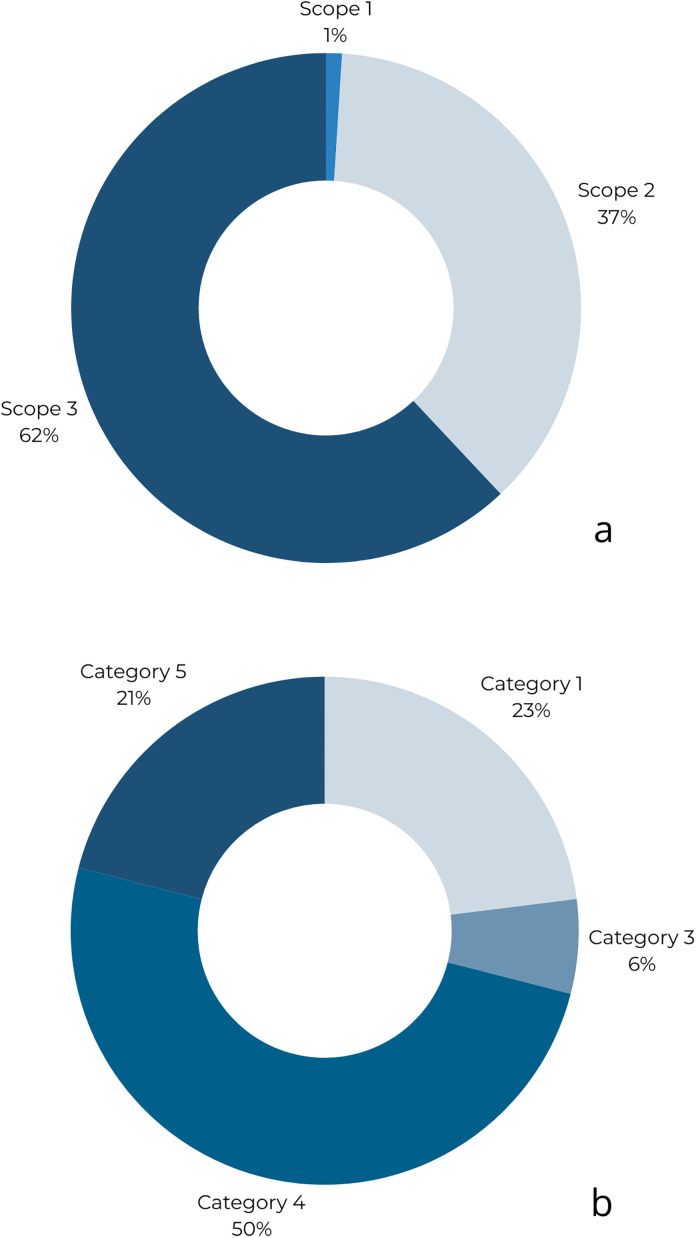
GHG emissions (as %) by Scopes (a) and composition of GHG emissions (as %) under Scope 3 (b) for Hotel «B».

The analysis shows distinct carbon-footprint profiles for the two facilities, with Scope-specific contributions differing in magnitude but pointing to the same hot spots: purchased energy (electricity/heat) and selected Scope 3 categories tied to procurement and waste. As summarized in Fig 3a and Fig 4a, direct emissions (Scope 1) are largely driven by stationary combustion where present, while Scope 2 reflects the carbon intensity of the grid supply. Scope 3 dominates in categories linked to food and consumables, confirming that decarbonization cannot rely on operational measures alone.

A closer look at Scope 3 confirms this pattern. Categories related to purchased goods and services (especially food) and waste generation contribute materially, whereas in-resort travel proved negligible in our setting (Fig 3b and Fig 4b). This This aligns with evidence that supply-chain choices—menu composition, sourcing, and material use—can shift footprints as much as metered energy savings. Accordingly, an effective mitigation portfolio must pair demand- and supply-side energy measures (efficiency, electrification where feasible, and cleaner electricity/heat) with procurement and waste strategies (menu shifts, portion control, avoided organics to landfill, and basic segregation for recyclables).

Furthermore, waste generation (Category 5) and the procurement of goods like food and cleaning suppliers (Category 1) are also substantial contributors, reflecting the resource intensity of hotel operations. Especially high contributors to these categories were meat products.

These finding suggests that a comprehensive decarbonization strategy for regional tourism in the context like that of the Pavlodar region must address emissions associated with transportation, nutrition and waste, in addition to emissions associated with electricity and heat.

When benchmarked against international data, the footprints of the Pavlodar businesses (227–529 tCO_2_-eq.) are comparable to similar enterprises in the tourism sector, although the underlying emission sources differ. For example, the average carbon footprint of a tourist entity in Europe, such as a mountain resort and hotel, varies between 100 and 500 tCO₂-eq. per year for small and medium-sized businesses [11]. The Kazakhstani businesses reflect the country’s reliance on fossil fuels for space and water heating, electricity, and transportation, while European counterparts have made a notable progress in accelerating the transition to renewable energy and improving energy efficiency resulting in emission reductions of 10–20% over the past 10 years. The calculated footprints of Kazakhstani businesses are notably lower than those of similar enterprises in other parts of Asia, such as India and Vietnam, where footprint can reach 700–800 tons of CO_2_-eq. per year due to a heavier reliance on coal and less mature low-carbon technologies [11]. However, some Asian countries are also vigorously developing strategic programs aimed at reducing emissions in tourism, including switching to solar and wind energy. Such actions imply similar rates of carbon footprint reduction to those of Kazakhstan.

International assessments indicate that roughly 90% of tourism-related GHG emissions arise from the transport of tourists (land, sea, and air) [3,21]. These data are valid for international tourism, where tourists travel long distances to holiday destinations (several thousand kilometers). In domestic tourism, such sources as energy supply to tourism sites and local services are most likely to prevail in the carbon footprint over tourist transportation [22]. This conclusion is also confirmed by our research on tourism businesses in Pavlodar region.

The decarbonization targets proposed in this study—50% absolute reduction by 2030 from the 2023 baseline with steady year-on-year improvements—are consistent with sectoral trajectories and broader net-zero commitments and are technically actionable through the combined levers above [23]. Real-world deliverability will depend on grid decarbonization, the pace of efficiency retrofits, and procurement policies (e.g., menu re-design and supplier standards) at the enterprise level.

Therefore, while the proposed targets meet international standards, their implementation must be adapted to the national context. The qualitative Scope 3 targets, focusing on developing the concepts for sustainable waste management, and sustainable nutrition and procurement by 2028, represent pragmatic initial steps to address the largest portion of the emissions. Beyond the environmental benefits, successfully implementing these plans can offer a significant competitive advantage. By transparently reporting on their climate action, these businesses can enhance their brand reputation and attract environmentally conscious tourists [24].

The results point to (i) the value of scope-resolved MRV at enterprise level, (ii) the need to synchronize facility plans with regional power-sector pathways, and (iii) the usefulness of procurement guidance (menus, supplier criteria, waste contracts) in small tourism businesses. These findings support target-setting and green-finance readiness for the Pavlodar tourism sector and can inform regional programs that link enterprise MRV with incentives for energy upgrades and circular procurement [25].

## 5. Conclusion

This study delivers the first scope-resolved carbon-footprint baseline for tourism businesses in Kazakhstan’s Pavlodar region, using two representative facilities as case studies. The inventories reveal distinct operational profiles but converging hot spots: (i) direct coal combustion where applicable (Scope 1), (ii) carbon-intensive grid electricity (Scope 2), and (iii) material Scope 3 categories linked to procurement and waste. Across both facilities, Scope 3 constitutes the largest share (57–62%), driven primarily by tourist transport, food purchases, and waste treatment, while Scope 1 and 2 magnitudes track energy carriers and operating seasons. These patterns clarify where mitigation can deliver the biggest and fastest gains.

Anchored in these findings, we propose a pragmatic decarbonization pathway aligned with SBTi ambition and Kazakhstan’s long-term strategies: absolute reductions of 15% by 2030, 25% by 2040, and 50% by 2050, complemented by carrier-specific targets—coal phase-out by 2050 and low-carbon electricity by 2035. The corresponding action set pairs demand- and supply-side energy measures (no-regret efficiency, electrification where feasible, and cleaner power/heat) with procurement and waste levers (menu re-design toward lower-carbon options and local sourcing, supplier criteria, avoided organics to landfill, basic segregation). Facility-level MRV closes the loop by turning one-off accounting into continuous management.

The work adds practical value on three fronts. First, it provides a replicable method—from system boundaries to factor selection—for small and medium hospitality operators in coal-reliant grids. Second, it prioritizes actions by quantifying scope/category contributions, enabling targeted investments and green-finance readiness. Third, it links enterprise practice to policy, showing how facility roadmaps depend on regional power-sector decarbonization and local circular-economy services (waste, suppliers).

Limitations and next steps follow directly. Our two-site baseline establishes order-of-magnitude results and action priorities, yet broader sampling is needed to represent the region’s diversity of business models and seasons. Future work will scale the MRV framework to a regional sector inventory, refine local emission factors, and assess nature-based offsets and other high-integrity mechanisms suitable for Pavlodar’s forest/forest-steppe landscapes—strictly as complements to deep reductions. Together, these steps can translate baselines into accountable progress toward Kazakhstan’s 2060 carbon-neutrality goal.

## References

[1] Intergovernmental Panel on Climate Change. Climate Change 2023: Synthesis Report. Contribution of Working Groups I, II and III to the Sixth Assessment Report of the Intergovernmental Panel on Climate Change; Geneva: IPCC; pp. 2023;35–115. doi: 10.59327/IPCC/AR6-9789291691647

[2] One Planet Network. The Glasgow Declaration: A Commitment to a Decade of Tourism Climate Action. https://www.oneplanetnetwork.org/sites/default/files/2022-02/GlasgowDeclaration_EN_0.pdf. 2022. Accessed 2025 February 8.

[3] Lenzen M, Sun Y-Y, Faturay F, Ting Y-P, Geschke A, Malik A. The carbon footprint of global tourism. Nature Clim Change. 2018;8(6):522–8. doi: 10.1038/s41558-018-0141-x

[4] Yessimova D, Faurat A, Belyi A, Yessim A, Sadykov Z. Environmental sustainability and carbon footprint of tourism: a study of a natural park in Northeastern Kazakhstan. Sustainability. 2025;17(4):1723. doi: 10.3390/su17041723

[5] Yessimova D, Faurat A, Belyy A, Yessim A, Novikova A, Olshanskaya M, et al. Assessment of the readiness of the tourism industry in the pavlodar region for the implementation of sustainable tourism. GTG. 2024;54(2 supplement):967–76. doi: 10.30892/gtg.542spl20-1271

[6] Yessim A, Shokhan R, Yessimova D, Faurat A, Safarov R, Sonko SM. Analysis of the economic state of the tourist industry in the pavlodar region (Kazakhstan). GTG. 2023;47(2):595–604. doi: 10.30892/gtg.47227-1059

[7] Puig R, Kiliç E, Navarro A, Albertí J, Chacón L, Fullana-I-Palmer P. Inventory analysis and carbon footprint of coastland-hotel services: a Spanish case study. Sci Total Environ. 2017;595:244–54. doi: 10.1016/j.scitotenv.2017.03.245 28384580

[8] Hu AH, Huang C-Y, Chen C-F, Kuo C-H, Hsu C-W. Assessing carbon footprint in the life cycle of accommodation services: the case of an international tourist hotel. Int J Sustainable Development World Ecol. 2015;22(4):313–23. doi: 10.1080/13504509.2015.1049674

[9] Bohdanowicz P, Martinac I. Determinants and benchmarking of resource consumption in hotels—Case study of Hilton International and Scandic in Europe. Energy and Buildings. 2007;39(1):82–95. doi: 10.1016/j.enbuild.2006.05.005

[10] Filimonau V, Dickinson J, Robbins D, Huijbregts MAJ. Reviewing the carbon footprint analysis of hotels: Life Cycle Energy Analysis (LCEA) as a holistic method for carbon impact appraisal of tourist accommodation. J Clean Prod. 2011;19:1917–30.

[11] World Travel & Tourism Council. A Net Zero Roadmap for Travel & Tourism: Proposing a New Target Framework for the Travel & Tourism Sector. London: World Travel & Tourism Council; 2021.

[12] EIB Project. Carbon Footprint Methodologies for the Assessment of Project Greenhouse Gas Emissions and Emission Variations. EIB Project. 2023.

[13] Intergovernmental Panel on Climate Change. IPCC Guidelines for National Greenhouse Gas Inventories. Geneva: Intergovernmental Panel on Climate Change. 2006. https://www.ipcc-nggip.iges.or.jp/public/2006gl/

[14] World Resources Institute, World Business Council for Sustainable Development. The Greenhouse Gas Protocol: A Corporate Accounting and Reporting Standard. Revised ed. Washington, DC: World Resources Institute. 2004.

[15] World Resources Institute, World Business Council for Sustainable Development. The greenhouse gas protocol: corporate value chain (scope 3) accounting and reporting standard/supplement to the ghg protocol corporate accounting and reporting standard. Washington, DC: World Resources Institute. 2011.

[16] World Resources Institute, World Business Council for Sustainable Development. The greenhouse gas protocol: Technical guidance for calculating scope 3 emissions/supplement to the corporate value chain (scope 3) accounting & reporting standard. Washington, DC: World Resources Institute. 2013.

[17] International Organization for Standardization. ISO 14064-1: Greenhouse gases. 2018.

[18] ghgprotocol.org [Internet]. Washington, DC: World Resources Institute and World Business Council for Sustainable Development; 2015 [cited 2025 Feb 08]. Available from: https://ghgprotocol.org/corporate-standard

[19] On Approval of the List of Benchmarks in the Regulated Sectors of the Economy. acting Minister of Ecology, Geology and Natural Resources of the Republic of Kazakhstan. 2021. https://adilet.zan.kz/rus/docs/V2100023621

[20] Science based targets. Science Based Targets initiative. https://sciencebasedtargets.org/resources/files/Net-Zero-Standard.pdf. 2024. Accessed 2025 February 8.

[21] Qureshi MI, Elashkar EE, Shoukry AM, Aamir A, Mahmood NHN, Rasli AMd, et al. Measuring the ecological footprint of inbound and outbound tourists: evidence from a panel of 35 countries. Clean Techn Environ Policy. 2019;21(10):1949–67. doi: 10.1007/s10098-019-01720-1

[22] Leal Filho W, Ng AW, Sharifi A, Janová J, Özuyar PG, Hemani C, et al. Global tourism, climate change and energy sustainability: assessing carbon reduction mitigating measures from the aviation industry. Sustain Sci. 2023;18(2):983–96. doi: 10.1007/s11625-022-01207-x 36105893 PMC9463512

[23] Li H, Sun Z, ChuanYu Y. Dynamic linkages between tourism development, renewable energy and high-quality economic development: Evidence from spatial Durbin model. PLoS One. 2024;19(2):e0295448. doi: 10.1371/journal.pone.0295448 38354176 PMC10866509

[24] Wagner N, Łapko A, Hącia E, Strulak-Wójcikiewicz R. Commitment to Sustainable Development Goals in marketing communications of ferry companies. PLoS One. 2024;19(12):e0312767. doi: 10.1371/journal.pone.0312767 39621671 PMC11611085

[25] He J, Zaman U. Sustainable sojourns: fostering sustainable hospitality practices to meet UN-SDGs. PLoS One. 2024;19(7):e0307469. doi: 10.1371/journal.pone.0307469 39046964 PMC11268582

